# Microfluidic Electroporation Arrays for Investigating Electroporation-Induced Cellular Rupture Dynamics

**DOI:** 10.3390/bios14050242

**Published:** 2024-05-11

**Authors:** Insu Park, Seungyeop Choi, Youngwoo Gwak, Jingwon Kim, Gyeongjun Min, Danyou Lim, Sang Woo Lee

**Affiliations:** 1 Department of Biomedical Engineering, Konyang University, Daejeon 35365, Republic of Korea; insu1023@gmail.com (I.P.);; 2 School of Biomedical Engineering, Korea University, Seoul 02481, Republic of Korea; sychoi0091@gmail.com; 3 Department of Biomedical Engineering, Yonsei University, Wonju 26493, Republic of Korea; 4 BK21 Four Institute of Precision Public Health, Korea University, Seoul 02841, Republic of Korea

**Keywords:** dielectrophoresis, electroporation, voltage loading rate, rupture energy barrier, critical pore radius

## Abstract

Electroporation is pivotal in bioelectrochemistry for cellular manipulation, with prominent applications in drug delivery and cell membrane studies. A comprehensive understanding of pore generation requires an in-depth analysis of the critical pore size and the corresponding energy barrier at the onset of cell rupture. However, many studies have been limited to basic models such as artificial membranes or theoretical simulations. Challenging this paradigm, our study pioneers using a microfluidic electroporation chip array. This tool subjects live breast cancer cell species to a diverse spectrum of alternating current electric field conditions, driving electroporation-induced cell rupture. We conclusively determined the rupture voltages across varying applied voltage loading rates, enabling an unprecedented characterization of electric cell rupture dynamics encompassing critical pore radius and energy barrier. Further bolstering our investigation, we probed cells subjected to cholesterol depletion via methyl-β-cyclodextrin and revealed a strong correlation with electroporation. This work not only elucidates the dynamics of electric rupture in live cell membranes but also sets a robust foundation for future explorations into the mechanisms and energetics of live cell electroporation.

## 1. Introduction

Cell membranes serve as essential barriers that ensure cellular integrity and homeostasis. The phenomenon of electroporation, where membranes are permeabilized using electric fields, holds great potential due to its transformative implications for cellular physiology and metabolic pathways. Notably, this process facilitates the swift movement of ions and molecules by creating transient or permanent aqueous channels in the membrane [1,2,3].

Electroporation can be categorized into reversible and irreversible types. Reversible electroporation is a temporary modification that enables the introduction of therapeutic agents without causing lasting cellular damage. This has been instrumental in gene therapy, where it aids the delivery of genes into cells, as well as in drug delivery, targeting diseases such as cancer [4,5]. In contrast, irreversible electroporation establishes permanent membrane pores that lead to cell death, such as apoptosis. This characteristic is particularly advantageous in cancer therapy, where electrical pulses can selectively target and destroy cancer cells while leaving healthy tissues unharmed [6,7].

To generate reversible/irreversible electroporation on the cell membranes, microfluidic techniques that significantly enhance the precisive manipulation of electroporation have been used for micro-scale cells, which is crucial for genetic modification in cellular therapy [8,9,10,11]. Traditional electroporation systems such as cuvette-based, plate-based, and in vivo electroporators have limitations in precision, automation, and system integration. Microfluidic-based electroporation devices address these issues by allowing precise control over the electric field and reducing adverse thermal effects that could damage cells [12]. These devices can be integrated with miniaturized electrodes to create uniform electric fields with lower voltages, improving transfection efficiency while maintaining high cell viability. Additionally, they enable superior heat dissipation and continuous flow, which requires the effective processing of large volumes of cells, even at a single-cell level. Various designs of microelectrode chips, such as micro-needles, micro-posts, nanopores, interdigitated electrodes, and microtrap arrays, have been developed [13,14,15]. These innovations allow for precise electroporation control, facilitating high-throughput analysis from multi-cell to single-cell levels. However, there remains a gap in the precise control of external forces required to analyze the dynamics of cell membrane disruption.

A deep understanding of the dynamics of cell membrane disruption during electroporation is crucial in deciphering the mechanism of pore formation. Numerous factors, encompassing the intensity and duration of the electric field, ambient temperature, and the intrinsic cell membrane properties, determine the energy essential for pore creation [16,17,18]. Central to this dynamic is the critical pore size, influenced by inherent cell features and the surrounding environment, and the critical energy threshold that destabilizes the cell membrane, leading to pore formation. Achieving a holistic grasp of the critical pore radius and energy barrier enables the refinement of electroporation conditions, resulting in enhanced membrane permeabilization and superior drug or gene delivery efficiency. This knowledge can also pave the way for the development of next-generation electroporation devices that boast heightened accuracy and performance.

A variety of techniques exist to investigate the dynamics of cell membrane rupture. Experimentally, fluorescence microscopy is often used, where pore formation is inferred from cellular dye uptake. Electrophysiology offers another avenue, gauging the electrical conductivity of the cell membrane in the electroporation process [19]. Although these experimental techniques excel in quantifying material transfer both intra- and extracellularly via electroporation and in assessing the penetration efficiency of target substances, they possess inherent limitations in the precise manipulation and evaluation of individual cells within the system. Theoretical studies have characterized cell rupture dynamics, with simulations detailing pore sizes and related parameters; however, there are limitations in implementing empirical models that embody the complex ecological system of the cell membrane in the electroporation process [20,21,22]. Notwithstanding these efforts, many investigations have predominantly focused on artificial membranes (e.g., giant unilamellar vesicles, dioleoyl-phosphatidylcholine (DOPC), 1-palmitoyl-2-oleoyl-phosphatidylcholine (POPC), and 1,2-dimyristoyl-sn-glycero-3-phosphocholine (DMPC)), and there remains an unmet need for experimental studies to investigate the electroporation dynamics on live cell membranes. Furthermore, the experimental development of the precise controls of external stimuli (e.g., voltage loading rate, electric field duration, and temperature), which can modulate cell rupture, is still challenging.

In this study, we present a new experimental method to determine the critical pore radius and energy barrier of the membrane rupture of live breast cancer cells on a microfluidic electroporation chip array. By measuring the rupture voltages of the individual cells under different applied voltage loading rates, we could determine the dynamics of electric cell rupture, including the critical pore radius and critical energy barrier, which were inferred using the Arrhenius model. Furthermore, we also determine the rupture dynamics with different membrane compositions by depleting the cholesterol in the membrane using methyl-β-cyclodextrin (MβCD). This study provides a new experimental approach in the field of electroporation and provides important information for understanding cell rupture mechanisms. This has the potential to significantly impact the development of cancer therapies as well as other areas such as gene therapy, vaccine development, and drug delivery.

## 2. Materials and Methods

### 2.1. Materials and Reagents

We used silicon dioxide wafers (i-Nexus, Inc., Seongnam, Republic of Korea) as the cell chip substrate and polydimethylsiloxane (PDMS), which consists of commercial Sylgard 184 Elastomer and its curing agents (Dow Corning, Midland, MI, USA), as the microfluidic chamber material. The microscope coverslip was purchased from SPL Life Sciences (Pocheon, Republic of Korea). For cell culture material, we used Dulbecco’s modified Eagle’s medium (DMEM, Cat. No. 11995-065, Gibco; Thermo Fisher Scientific Inc., Waltham, MA, USA), fetal bovine serum (FBS, Cat. No. 16000-044, Gibco, Thermo Fisher Scientific Inc., Waltham, MA, USA), penicillin-streptomycin (PS, Cat. No. 15140-122, Gibco), and Trypsin-EDTA solution (0.25%, Cat. No. 25200-072). The MβCD (Cat. No. C4555-1G) powder was purchased from Sigma-Aldrich; Merck KGaA (Darmstadt, Germany). The dielectrophoretic (DEP) solution consisted of sucrose powder (8.6% *w*/*w*; Cat. No. SB0498, Bio Basic Inc., Markham, ON, Canada), D-glucose powder (0.3% *w*/*w*; Cat. No. GB0219, Bio Basic Inc.), and bovine serum albumin powder (BSA; 1.0 mg/mL, Bovogen Biologicals, Essendon, VIC, Australia) in deionized water (water purification system, Sartorius AG, Gottingen, Germany). Phosphate-buffered saline solution (PBS; 0.2% *v*/*v*, Cat. No. 20012-027, Gibco) was used to adjust the conductivity of the DEP solution to 60 μS/cm.

### 2.2. Fabrication of Microfluidic Electroporation Array Chip

The electrode configuration for the cell chip underwent a sequential fabrication process. The oxide/silicon wafer substrate was prepared with solvent (acetone 20 min, methanol 20 min) and piranha (H_2_SO_4_:H_2_O_2_ = 3:1, 25 min) cleaning. The photoresist (PR) was deposited by spin coating to 1 μm thickness. UV light exposures were used to develop a 30/10 μm (electrode width/electrode gap) interdigitated array (IDA) pattern using a photomask and developer. The electrode was deposited in a 0.1 μm chromium layer on the wafer using a thermal evaporator and PR layer elimination of the lift-off effect to gain an IDA electrode. To reduce electro-hydrodynamic effects such as alternating current (AC)-electro-osmosis and electrothermal effects, a 0.8 μm SiO_2_ insulating layer was deposited using plasma-enhanced chemical vapor deposition. To segregate cells individually on the chip via DEP force, 20 μm diameter circular regions resembling cells were etched on the SiO_2_ layer through conventional photolithography and wet etching processes. Before injecting cells into the chip for DEP experiments, the chip was cleaned using a piranha solution (H_2_SO_4_:H_2_O_2_ = 1:1) for 3 min and combined with the 5 mm diameter polydimethylsiloxane (PDMS) hole reservoir, which was prepared by mixing the PDMS elastomer and the PDMS curing agent in a 10:1 ratio and curing in an oven at 60 ℃ for more than 5 h.

### 2.3. Cell Preparation

In this study, we cultured the human breast cancer cell line MCF-7 in DMEM, supplemented with 10% FBS and 1% PBS. Cells were seeded in a 100 pi dish at a density of 1 × 10^5^ cells and cultured for 48 h under standard conditions (37.5 °C, 5% CO_2_, and a humidified atmosphere). The cells were then washed with fresh cell culture medium, treated with 0.25% Trypsin/Ethylenediaminetetraacetic acid (EDTA), and subsequently seeded in 6-well plates with 2 mL of new medium at a density of 2 × 10^5^ cells/mL for 24 h. These cells were harvested on a 6-well plate using Trypsin/EDTA solution using the above cell detachment technique before being injected into the DEP chip. The cell medium with each harvested cell line was replaced with the DEP experimental solution, yielding a density of approximately 200 cells/μL. For cholesterol depletion from cell membranes using MβCD, a 37.8 mM-MβCD solution in PBS was first diluted in DMEM to concentrations of 2.5 mM and 5 mM. This solution was added to cells cultured for 24 h in a 6-well plate. After 2 h of incubation, the MβCD-treated cells were washed twice with fresh serum-free DMEM and harvested as described previously.

### 2.4. Setup of Measurement System

**Figure 1:**
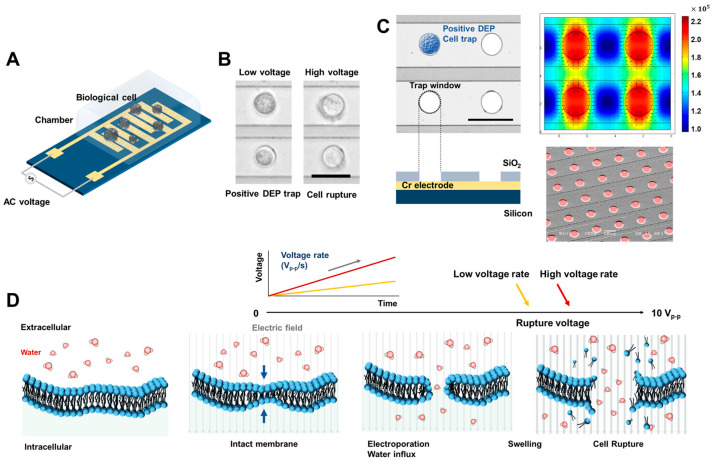
Designs for an electroporation chip. (**A**) Schematic diagram of an electroporation chip. (**B**) Microscope images of cell rupture by electroporation under low and high AC voltage conditions. Scale bar, 30 μm. (**C**) The microscope and SEM images of a fabricated electroporation chip array and corresponding non-uniform electric field distribution on the chip, induced by biological cell trap and electroporation. Scale bar, 30 μm. (**D**) The sequence of lipid bilayer formations of the intact membrane and electroporation process under a high AC electric field leads to cell rupture. The cell rupture moment depends on the applied voltage loading rates.

After the harvested cell solution was injected into the PDMS reservoir, the cell chip was sealed using a coverslip. It was then placed on the custom probe station (Modusystems Inc., Hanam, Republic of Korea) and fixed strongly with a vacuum pump. After contacting a microprobe with the arbitrary function generator (NI PCI-5421, National Instrument, Austin, TX, USA) connected to the electrode pad on the chip (Figure 1A), an AC sinusoidal signal was applied. The electricity-induced real-time cell movement and shape (Figure 1B) were observed in the top-view via a microscope that connected the charge-coupled device (CCD, Motionscope M3, Redlake, San Diego, CA, USA). This top-view image was recorded relative to the AC frequency or AC voltage increase, which is controlled by a custom LabVIEW-based automated system (National Instruments, Austin, TX, USA). The detailed automated measurement system was described in our previous studies [23,24,25].

### 2.5. Experimental Process

Before observation of the electrophoresis-induced cell rupture event, the dielectric property of the living cells (i.e., DEP crossover frequency) was examined to not only measure the membrane capacitance, but also to distinguish living and dead cells. Thus, we designed the input signal sequence to measure DEP crossover frequency and rupture voltage in the same environment, including cells, sample solution, and chip. First, DEP crossover frequency was measured by determining the change in DEP-force-induced cell direction by applying AC frequency modulations from 1 to 41 kHz (a more detailed measurement was described in our previous reports [25]). Subsequently, for the measurement of critical voltage, the input voltage is controlled in two steps: (1) Movement of the cells from the negative DEP trap position to the positive DEP trap position for rupture performance; and (2) constant frequency at 41 kHz and changing input voltage linear increase from 2 V_peak-to-peak(p-p)_ to 10 V_p-p_ at 0.05 V/s for 200 s, as in Figure 1D. These steps compose one set of experiments and are controlled by a LabVIEW-based automated DEP system. We utilized the NI-PCI-5421 as a sine wave function generator operating at a sampling rate of 108 samples per second. The input voltage was incrementally increased from 2 Vp-p to 10 V_p-p_ at rates of 0.003, 0.006, 0.012, 0.025, 0.05, and 0.1 V_p-p_ per second, respectively, while maintaining an AC frequency of 41 kHz. The frequency and amplitude of the applied voltage were monitored in real-time using an oscilloscope.

### 2.6. Simulation of Electric Field Strength

We conducted a simulation of the applied electric field in our setup using a finite element method (FEM) tool, COMSOL Multiphysics version 6.0. The three-dimensional electrode designs are depicted in Figure 1B. We used the AC/DC electrostatic model to model electric fields within dielectric substances. Leveraging the symmetry of our electroporation chip, we crafted a pair of electrodes, each featuring four circular trapping zones. To ensure the precision of the FEM solver, the mesh resolution was fine-tuned to surpass the smallest scale of particle movement.

#### 2.6.1. Calculation of Electric Tension

Electric tension is a key parameter to understanding the rupture mechanisms of the cell, which play an important role in determining the permeabilization of the cell membrane during cell rupture. The critical electric tension (CET) is the minimum electric field strength on the cell membrane required to create permeability for efficient transfection of foreign material, such as DNA or RNA, into the cell. Therefore, exceeding CET during electroporation can lead to cell rupture and osmotic lysis. To calculate CET from the measured rupture voltage, the membrane capacitance of the cell ($C_{mem}$) is calculated first, as in Equation (1) [26],

(1) $$C_{mem}=\frac{\sqrt{2}\sigma_{med}}{2\pi rf_{co}}$$

where $f_{co}$ is the crossover frequency of the cell, which was measured experimentally in our previous study [25]; r is the cell radius; and $\sigma_{med}$ is medium conductivity. In an AC electric field, transmembrane potential (TMP, $U_{tmp}$), which refers to the voltage difference between the inside and outside of a cell membrane, is calculated using $C_{mem}$ as in Equation (2) [27].

(2) $$U_{tmp}=\frac{1.5Er\cos\theta }{1+i\left(2\pi f\right)rC_{mem}\left(x_{cyt}+\frac{x_{med}}{2}\right)}$$

where f is the applied frequency (41 kHz in this study), $x_{cyt}$ is cytoplasm resistivity (reciprocal of cytoplasm conductivity), and $x_{med}$ is medium resistivity. TMP is maximized when cosθ is ~1. E is the applied electric field strength determined by the numerical simulation using COMSOL 6.0 software. The extraction point of E is defined as the cell membrane position closest to the electrode, and the applied voltage is considered the measured rupture voltage. At cell rupture, the TMP reaches its critical point. As a result, the membrane potential is destabilized, leading to a rapid and uncontrolled release of ions and other cellular contents. As the cell membrane is considered a capacitor model, the electrical energy is accumulated in the cell membrane and can be converted to mechanical energy (e.g., electrical tension) to generate a permanent pore in the cell membrane [18]. Therefore, the critical electrical tension ($\sigma_{c}$) generated by the critical TMP is described in Equation (3),

(3) $$\sigma_{c}=\frac{1}{2}C_{LW}{U_{tmp}}^{2},\;C_{LW}=C_{mem}\left\{\frac{\left(\frac{\varepsilon_{med}+\varepsilon_{cyto}}{2}\right)}{\varepsilon_{mem}}-1\right\}$$

where $C_{LW}$ is the change of capacitance as water displaces the lipids to form a pore, and $\varepsilon_{mem}$ and $\varepsilon_{cyto}$ are the dielectric permittivity of the membrane and the cytoplasm, respectively [28,29].

#### 2.6.2. Determination of Critical Energy Barrier and Pore Radius of Cell Rupture

To calculate the critical energy barrier and pore radius, an Arrhenius model elucidating the exponential relationship between the electrical field strength applied to a cell and the resulting rate of membrane rupture is used. The lifetime of the pore is described by Equation (4),

(4) $$\tau_{0}=\frac{1}{k_{0}}=\frac{1}{v_{0}}e^{\frac{W_{0}}{k_{B}T}}$$

where $k_{0}$ is the dissociation rate constant in the absence of applied force, $v_{0}$ is a pre-exponential factor (frequency factor ~1013 s^−1^), $k_{B}$ is the Boltzmann constant, *T* is absolute temperature, and $W_{0}$ is an energy barrier. Under external energy applied to the reaction, the Bell model [30,31] can be applied to Equation (4) as in Equation (5),

(5) $$\tau_{bell}=\frac{1}{v_{o}}e^{\frac{W_{0}-Fx_{b}}{k_{B}T}}=\tau_{0}e^{\frac{-Fx_{b}}{k_{B}T}}$$

where F is the external force for the reaction, and $x_{b}$ is the reaction coordinate, which means the size of the membrane pore during cell rupture in this study. Therefore, Equation (5) can be applied to the mechanical force ($F_{max}$) generating the cell rupture and force loading rate ($\dot{F}$) equation,

(6) $$F_{max}=\frac{k_{B}T}{x_{b}}ln\frac{\dot{F}x_{b}}{k_{B}Tk_{0}}$$

In this study, mechanical force is the electrical tension ($\sigma_{e}$) derived from the measured rupture voltages, the force loading rate is the electrical tension rate ($R\sigma_{e}$), and $x_{b}$ is the pore radius ($\pi r^{2}$). Corresponding to Equation (5), the Bell model in cell rupture dynamics can be expressed as Equation (7).

(7) $$\tau_{bell}=\frac{1}{v_{0}}e^{\frac{W_{o}-0.5C_{LW}U^{2}\pi r^{2}}{k_{B}T}}=\tau_{0}e^{\frac{-0.5C_{LW}U^{2}\pi r^{2}}{k_{B}T}},\;\tau_{0}=\frac{1}{v_{0}}e^{W_{0}}$$

Therefore, based on Equations (6) and (7), CET is generalized as in Equation (8).

(8) $$\sigma_{e}=\frac{k_{B}T}{\pi r^{2}}ln\frac{R\sigma_{e}\pi r^{2}}{k_{B}Tk_{0}}$$

## 3. Results and Discussion

### 3.1. Understanding Electroporation-Induced Cell Rupture Dynamics

**Figure 2:**
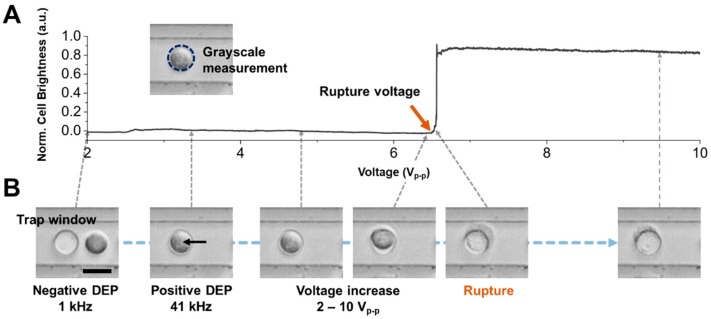
Characterization of the rupture voltage of cells on an electroporation chip array. (**A**) Measured grayscale intensity data on the trap window region as the applied AC voltage increases. The grayscale value is increased when the cell rupture occurs. The applied frequency is 41 kHz, trapping the cell on the trap window by positive DEP force. (**B**) Microscope images of cell rupture events at 2, 3.2, 4.8, 6.3, 6.5, and 9.5 V_p-p_. The cell is trapped in a negative DEP trap region at 1 kHz and a positive DEP trap region called a trap window at 41 kHz. The cell is deformed and ruptured when the applied voltage increases over 6.5 V_p-p_. Scale bar, 20 μm.

Our microfluidic electroporation chip is depicted in Figure 1A. The IDA electrode design is a conventional configuration that is widely used in applications such as dielectrophoresis-induced microparticle sorting. This configuration is particularly advantageous for observing the movement and morphology of hundreds of cells simultaneously due to its ability to provide high uniformity across the electrode area. To transmit external electrical energy to living cell membranes, the chip interfaces with a micromanipulator probe. This probe transmits AC sinusoidal signals, responsible for cell trapping and electroporation by DEP, from an electric signal generator. To closely monitor the morphology of electroporated cells, our chip is mounted onto a bright-field digital microscope (Figure 1B). This setup captures top-view image sequences via an automated LabVIEW-based measurement system. This system synchronizes the recording and signal generation modules (detailed in Materials and Methods 2.4). When the voltage is applied to the chip, the most intense electric field gradient is observed centrally within the circular zones and on the boundaries of the electrodes. The least intense region is the center point between two adjacent windows, as seen in Figure 1B. If a dielectric particle such as a cell has [(ω)] > 0, it moves toward the area with the highest electric field gradient, as shown in Figure 2B (termed a positive DEP trap). The opposite behavior is demonstrated in Figure 2B and is referred to as a negative DEP trap. We previously used an electrode configuration (illustrated in Figure 1C) that efficiently manipulated hundreds of cells simultaneously [23,25] and the reproducibility test of the chip is also in Supplementary Materials Figure S1. This configuration enhanced single-cell sorting accuracy. Notably, the majority of sorted cells experienced similar electric field distributions due to electrokinetic force. Such a consistent field distribution allows the statistical observation of the electroporation phenomenon, triggered when critical hyperpolarization thresholds are surpassed for individual cells [25].

When a cell is subjected to an electric field, its TMP—the voltage differential across its membrane—is altered. The electroporation phenomenon stems from this TMP variation, with the underlying mechanism depicted in Figure 1D. Initially, the altered TMP serves as a driving force, prompting water molecules, DNA, proteins, ions, and other polar entities inside and outside the cell membrane to converge. This process leads to increased lateral mechanical pressure. As the external electric field intensity escalates, the permeability of the cell membrane increases, facilitating the entry of DNA or other molecules into the cell. In the final stage, if the pores expand beyond a certain critical radius due to the augmented TMP, the osmotic pressure inside the cell shifts. This can cause the cell to swell, or in extreme cases, rupture. It is important to emphasize that rupture dynamics, such as the critical energy barrier and pore radius, are significantly influenced by the energy applied externally. Hence, our study also encompassed measurement of the critical rupture voltages of cells achieved by modulating the rate of voltage escalation.

### 3.2. Determination of Cell Rupture Voltages

Figure 2 provides an illustrative analysis of a single MCF-7 cell subjected to AC signal modulation. As discussed earlier, MCF-7 cells resting randomly on the chip substrate are drawn to the interspace between adjacent trap window electrodes, guided by the direction of negative dielectrophoretic force (set at 1 kHz, 2 V_p-p_). Once most of the cells are positioned in the observed top-view region, the AC frequency is reduced from 41 kHz to 1 kHz. The crossover frequency at which the cell is released from the trapping region is then measured, leveraging the image processing analysis methodology described in our prior work [25]. Subsequently, AC voltage was increased from 2 V_p-p_ to 10 V_p-p_. Based on our previous work, under these frequency and amplitude conditions, the Joule heating occurring within the chip is negligible [23,24,25]. The change in cell shape is abruptly initiated toward the transparent shape (Figure 2B, “Rupture” image). Figure 2A shows the brightness distribution within this cell region depending on voltage modulation. The level of the brightness signal also appeared to shift suddenly, which is attributed to the irreversible electroporation phenomenon [32]. We measured the rupture voltage when the cell brightness started to increase using image correlation analysis. The cell brightness in a 3-standard deviation voltage over average rupture voltage (e.g., 2 V_p-p_) remained unchanged, which indicates that the cell brightness signal could clearly show the cellular electroporation phenomenon.

### 3.3. Cell Rupture at Various Voltage Loading Rates

**Figure 3:**
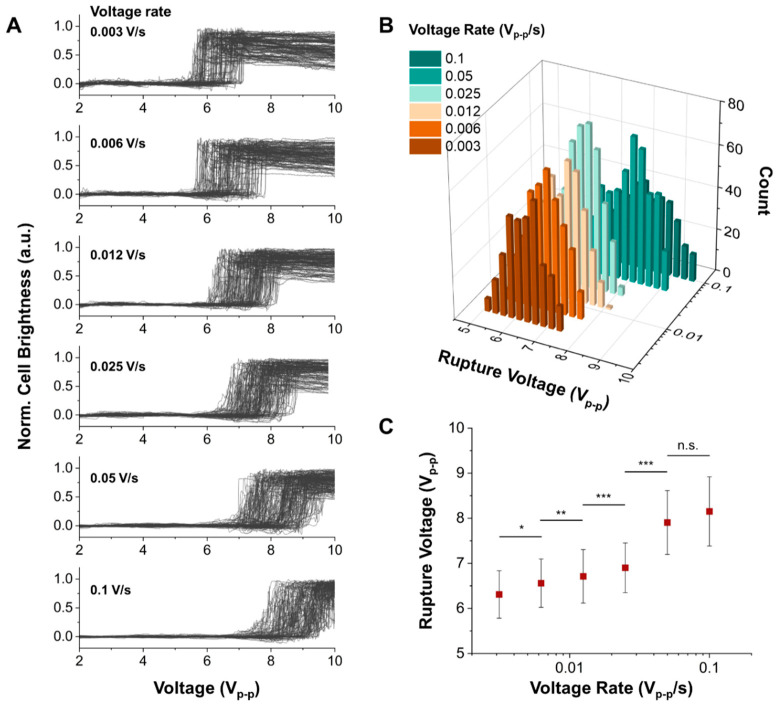
Cell rupture events in various voltage loading rates. (**A**) Grayscale data correspond to the deformation and rupture of the cell with the different voltage loading rates measured as a function of applied voltage. The applied frequency is 41 kHz. (**B**) Histogram representing the number of ruptured cells as a function of rupture voltage with different voltage loading rates. (**C**) Mean rupture voltage vs. voltage loading rate. n.s.; not significant. Statistical test; *p*-value, * *p* < 0.05, ** *p* < 0.005, *** *p* < 0.0005.

To measure the dynamic response of cell rupture by modulating the voltage loading rate, we measured the rupture voltages of MCF-7 cells with different voltage loading rates from 0.003 V/s to 0.1 V/s. As shown in Figure 3A, the rupture moment of more than 200 cells was simultaneously measured on a chip with different voltage loading rates. To determine the precise rupture moment of each cell, a microscope camera captured the images with 10 frames/s. In each image sequence corresponding to the applied voltages, the change in cell brightness, defined as the cell rupture moment, was analyzed using the image processing method developed in our previous work [23]. The population of the cell rupture moment was shifted when the applied voltage loading rate increased. The histogram in Figure 3B consolidates these observations, mapping out the rupture voltages of MCF-7 cells against their corresponding voltage loading rates. A statistical difference occurs in the rupture voltages along 0.003, 0.006, 0.012, 0.025, and 0.05 V_p-p_/s, as shown in Figure 3C. However, there is no significant difference between 0.05 and 0.1 V_p-p_/s, suggesting that our maximal voltage loading rate (0.1 V_p-p_/s) suffices in measuring the saturation point of cell rupture dynamics under heightened external energy conditions. The dynamics of cell rupture in response to the electric field condition is a complex and multifaceted process related to the lifetime of pores and the charging process of the membrane capacitor, which represents the change in TMP when reaching the critical values generating irreversible electroporation. The Arrhenius model (Materials and Methods. 2.6.2) describes the relationship between the rate of cell rupture and the energy barrier required to initiate the reaction. In cell rupture, the energy barrier refers to the critical energy required to disrupt the cell membrane and create a pore. Under the electric field modulation, such as voltage loading rates, the Bell model can be adapted to consider the external energy stimuli. This model suggested that the cell rupture was proportional to the natural logarithm of the loading rate, which is closely related to the voltage loading rate in this study. It can explain how the rupture voltage increases as the voltage loading rate increases in Figure 3C. To determine the critical energy barrier and pore radius in cell rupture, the critical electrical tension ($\sigma_{e}$) and electrical tension rate ($R\sigma_{e}$) were derived from the measured rupture voltages and voltage loading rates in Figure 3C (Materials and Methods. 2.6.1). Notably, it shows the linear relationship between the critical electrical tension and electrical tension rate, which follows the Bell model equation. Using this theoretical model and the linear slope (~0.001), we determined experimentally that the critical energy barrier (ΔG) and critical pore radius (*r*) of MCF-7 by electroporation are 13.59 and 1.07 nm, respectively. This result is similar to the estimation by numerical simulation [18,22,33].

### 3.4. Impact of Cholesterol Composition on Cell Rupture Dynamics

**Figure 4:**
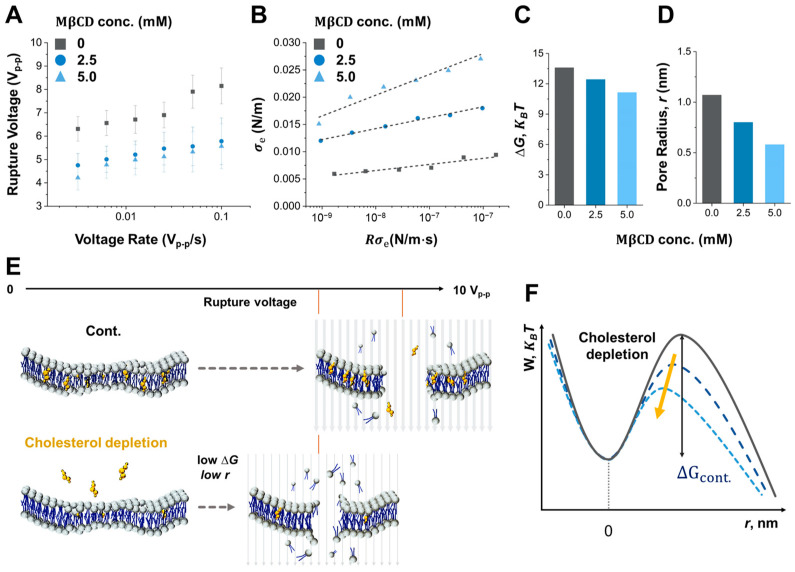
Free energy of the cell rupture with different cholesterol concentrations in the membrane. (**A**) Result of rupture voltage as a function of voltage loading rate with different MβCD concentrations. (**B**) Results of critical electrical tension ($\sigma_{e}$) of cell rupture as a function of electrical tension rate ($R\sigma_{e}$) with different MβCD concentrations. Electrical tension and electrical tension rate are calculated from measured rupture voltages and applied voltage loading rates in (**A**), respectively. (**C**) The result of free energy (increment cap G, KBT) and (**D**) pore radius (r, nm) correspond to the M beta CD concentration Energy and pore radius values are calculated from (**B**) in conjunction with the Bell equation. (**E**) Schematic illustration of the electroporation formation of Lipid bilayer at low cholesterol concentration. (**F**) Schematic illustration of the energy pathway of cell rupture dependent on the cholesterol concentration in a membrane.

Another critical factor affecting cell rupture dynamics is the composition of the cell membrane. At its core, the cell membrane is a structured lipid bilayer amalgamating phospholipids, cholesterol, and an assortment of other lipids and proteins. This cell membrane composition influences its mechanical properties, including stiffness and resistance to cell rupture. In understanding the defense mechanisms against external perturbations, especially those triggered by electric fields, the mechanical integrity of the cell membrane is crucial. The Bell model, which incorporates this inherent stability along with the applied external energy, provides a comprehensive forecast of the dynamics underlying pore creation. In this study, the applied voltage loading rate is an external stimulus in determining both the speed and extent of pore development on the cell membrane. As this process advances, fluctuations in membrane mechanical properties emerge as crucial feedback mechanisms, elucidating the level of cellular electroporation and rupture. Both the critical energy barrier and the pore size set the conditions essential for the efficacy of irreversible electroporation [34,35]. With this understanding, we used different concentrations of MβCD (0 mM, 2.5 mM, and 5 mM) to deplete the cholesterol from the MCF-7 cell membrane systematically, which showed significant differences in the cholesterol contents of the membrane used in our previous study [25]. Figure 4A is the measured rupture voltages as a function of voltage rate for each concentration of MβCD. The critical electrical tension ($\sigma_{e}$) based on the results in Figure 4A is also calculated in Figure 4B. As a result, the linear slope at each MβCD concentration is 0.001, 0.002, and 0.004, respectively. The critical energy barriers and pore radius of MCF-7 cells treated by 0 mM, 2.5 mM, and 5 mM MβCD, which are calculated by the Bell model and the linear slopes in Figure 4B, which are 13.59, 12,4, 11.1, and 1.07 nm, 0.80 nm, 0.58 nm, respectively (Figure 4C,D). It is well known that the cholesterol content of cell membranes plays a crucial role in determining the mechanical stability and susceptibility to rupture by decreasing lipid packing. The results of our study indicate that the cholesterol content of the cell membrane influences cell rupture. More importantly, mechanical stability and resistance as the cholesterol contents of cell membranes are also affected by the loading stress generated by the variation of voltage increase rate, as shown in Figure 4A,D. When cholesterol in a cell membrane is depleted, cell rupture by electroporation occurs more easily since the critical energy barriers and pore size, which correlate to the dynamics of loading stress to the cell membrane, are decreased, as shown in Figure 4E,F. However, this relationship between cholesterol content and cell membrane stability is not linear. At high levels, cholesterol can disrupt the normal lipid packing of the membrane and make it more prone to mechanical failure, leading to increased cell rupture. Further research is needed to fully understand the mechanisms behind the cholesterol effects that generate cell rupture by electroporation as a function of the voltage loading rate.

## 4. Conclusions

In this study, we present experimental methods and analysis for determining the critical energy barrier and critical pore radius in cell rupture by electroporation on a microfluidic electroporation chip array, which enables us to measure hundreds of cell rupture events simultaneously. Precise control of the voltage loading rate allows the determination of the dynamic responses in cell rupture. Based on this experimental approach, the critical energy barrier and critical pore radius in live cell rupture are analyzed using the Bell model. It is validated that the required energy and critical pore radius decrease when the cholesterol is depleted in the cell membrane since it lowers the mechanical stability and increases susceptibility to rupture. In addition, the loading stress that is generated by that voltage loading rate also affects mechanical stability and resistance, as well as the cholesterol content of cell membranes. The experimental study of cell rupture dynamics in live cells demonstrated here is expected to be useful for a better understanding of the dynamics of cell rupture in response to external stimuli and also to provide practical information in bioelectrochemistry fields such as drug delivery, gene therapy, and cell biology with electroporation.

This paper presents an initial experimental approach for analyzing cell rupture dynamics in live cells. However, optimization studies on the chip structure are necessary for researching rupture dynamics in various types of cells, including adherent and suspension cells. Our current method, an automated cell tracking system operating at 2 s per frame, is suboptimal for real-time analysis. To address this limitation, we are developing artificial intelligence-based cell-tracking methods to enable real-time monitoring of live cell rupture dynamics [36]. Moreover, complex integrating technologies that can apply diverse loading stresses are necessary for a comprehensive understanding of cell rupture dynamics under varying physicoYchemical conditions. Enhancing observation precision through the integration of transparent substrates and electrodes will improve the monitoring of material transport across cell membranes, thereby facilitating more precise therapeutic interventions targeting cell rupture.

## Data Availability

You can contact the corresponding author as yusuklee@yonsei.ac.kr.

## Supplementary Materials

The following supporting information can be downloaded at: https://www.mdpi.com/article/10.3390/bios14050242/s1, Table S1. Abbreviation and Nomenclature. Table S2. Comparison between conventional techniques that induce cell perforation and this work. Figure S1. Reproducibility test of the DEP chip. Each test was conducted using the same DEP device with a voltage rate of 0.1 Vp-p/s. All data showed no significant variations, refs. [6,37,38,39,40,41,42,43,44,45,46,47,48,49,50,51,52].
