# Supplementary material for: Microfluidic Electroporation Arrays for Investigating Electroporation-Induced Cellular Rupture Dynamics
Source: Biosensors (Basel). 2024 May 11;14(5):242. doi: 10.3390/bios14050242 (PMC11118139; doi:10.3390/bios14050242)
Supplement: Supplementary file 1 [file biosensors-14-00242-s001.zip › biosensors-2921325-supplementary.pdf]

Supplementary Materials Appendix for

Microfluidic Electroporation Arrays for Investigating Electroporation-Induced Cellular Rupture Dynamics

Insu Park[1,†], Seungyeop Choi[2,3,4†], Youngwoo Gwak[3], Jingwon Kim[3], Gyeongjun Min[3], Danyou Lim[1], and Sang Woo Lee[3,*]

[1]Department of Biomedical Engineering, Konyang University, Daejeon, 35365, Republic of Korea

[2]School of Biomedical Engineering, Korea University, Seoul 02841, Republic of Korea

[3]Department of Biomedical Engineering, Yonsei University, Wonju, 26493, Republic of Korea

[4]BK21 Four Institute of Precision Public Health, Korea University, Seoul 02841, Republic of Korea

*****Correspondence: yusuklee@yonsei.ac.kr

†These authors contributed equally to this work.

Supporting Table

Table S1. Abbreviation and Nomenclature

**DOPC**	dioleoyl-phosphatidylcholine
**POPC**	1-palmitoyl-2-oleoyl-phosphatidylcholine
**DMPC**	1,2-dimyristoyl-sn-glycero-3-phosphocholine
**MβCD**	methyl-β-cyclodextrin
**PDMS**	polydimethylsiloxane
**DMEM**	Dulbecco's modified Eagle's medium
**IDA**	interdigitated array
**EDTA**	Ethylenediaminetetraacetic acid
**AC**	alternating current
**FEM**	finite element method
**CET**	critical electric tension
**TMP**	transmembrane potential
C_{mem}	membrane capacitance of the cell
r	cell radius
σ_{med}	medium conductivity
f	the applied frequency
E	the applied electric field strength
σ_c	critical electrical tension
C_{LW}	the change of capacitance as water displaces the lipids to form a pore
τ_0	The lifetime of the pore
k_0	dissociation rate constant in the absence of applied force
V_0	a pre-exponential factor (frequency factor ~10^{13} s^{-1})
k_B	Boltzmann constant
T	absolute temperature
W_0	energy barrier
**F**	the external force for the reaction
x_b	the reaction coordinate, which means the size of the membrane pore during cell rupture
F_{max}	the mechanical force
σ_e	electrical tension
$R\sigma_e$	the electrical tension rate
ω	angular frequency

Table S2. Comparison between conventional techniques that induce cell perforation and this work.

Method		External energy source	Target	High-throughput analysis	Single cell analysis	Rupture Dynamics $\Delta w, KBT$ Pore radius (nm)	Ref.
**Molecular dynamics (MD) simulation**		Mechanical tension	Lipid bilayer	No	No	10 - 40, K_BT 1-1.45 nm	[18,28, 37,38]
**Micropipette aspiration**		Mechanical pressurization	Lipid bilayer	No	Yes	10 - 19.8, K_BT 2 - 3.5 nm	[39-41]
**Micro-indenter, AFM tip**		Mechanical compression	Adherent cell Lipid bilayer	No	Yes	N.A.	[42,43]
**patch-clamp amplifier**		Electroporation	Adherent/suspension cell	No	Yes	25, K_BT 0.5 nm	[44,45]
**Microfluidics-based Electroporation**	**Static**	Electroporation (Micro-/Nanopillar Arrays Microbead Packing)	Adherent cell	Yes	Yes	N/A	[6,46-49]
	**Continuous**	Electroporation (Channel Constriction Microelectrode array)	Adherent/suspension cell	Yes	No	N/A	[6,49-52]
***This work**		Electroporation (Dielectrophoresis)	Adherent/suspension cell	Yes	Yes	13.59, K_BT 1.07 nm	

Supporting figure

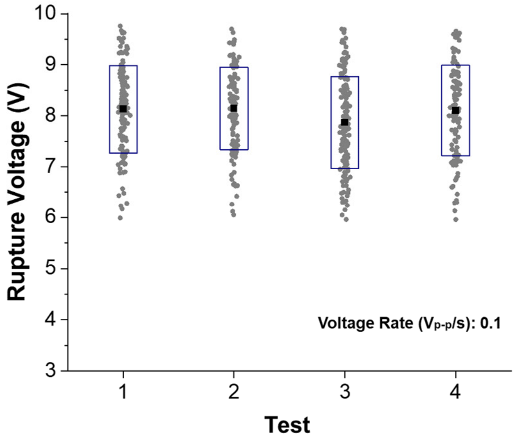

Figure S1. Reproducibility test of the DEP chip. Each test was conducted using the same DEP device with a voltage rate of 0.1 Vp-p/s. All data showed no significant variations.
